# Unmasking Carcinoid Syndrome in a Chronic Obstructive Pulmonary Disease (COPD) Patient: A Rare Presentation with Wheezing and Angioedema

**DOI:** 10.7759/cureus.61321

**Published:** 2024-05-29

**Authors:** Mesrop Aleksanyan, Sindhu Chadalawada, Knkush Hakobyan, Xuebin Yang, Emily Chen

**Affiliations:** 1 Internal Medicine, Capital Health Regional Medical Center, Trenton, USA; 2 Pathology and Laboratory Medicine, Capital Health Regional Medical Center, Trenton, USA; 3 Hematology and Medical Oncology, Capital Health Regional Medical Center, Trenton, USA

**Keywords:** unresponsive wheezing, metastatic carcinoid tumor, metastatic liver tumor, facial angioedema, wheezing as a main symptom, gastrointestinal carcinoid tumor

## Abstract

Carcinoid syndrome is a rare condition resulting from neuroendocrine tumors (NETs) that secrete vasoactive substances like serotonin. This report describes the case of a 61-year-old man with a history of chronic obstructive pulmonary disease (COPD) and hypertension who presented with new-onset angioedema, loss of consciousness, and a fall. He had been treated for COPD exacerbations during ER visits without improvement and was unaware of a prior mesenteric carcinoid tumor diagnosis from 2012. The next emergency evaluation revealed significant airway and facial edema necessitating intubation. Imaging and biopsy identified a well-differentiated grade 1 NET with extensive liver metastases. Laboratory tests showed elevated levels of serum serotonin, chromogranin A, and 24-hour urine 5-hydroxyindoleacetic acid (5-HIAA). Post-discharge, a PET scan confirmed metastatic lesions primarily in the liver and small bowel, with an unresectable mesenteric mass. The patient was treated with lanreotide and became symptom-free. This case underscores the need to consider carcinoid syndrome in patients with COPD presenting with unexplained respiratory symptoms, as timely diagnosis and treatment can significantly enhance patient outcomes.

## Introduction

Carcinoid tumors are a heterogeneous group of neuroendocrine neoplasms that can arise anywhere in the body, but most commonly occur in the gastrointestinal (GI) tract. Carcinoid syndrome (CS) is a constellation of clinical symptoms caused by the release of vasoactive substances and hormones from carcinoid tumors and can include wheezing, shortness of breath, and angioedema [1].

Wheezing is a relatively rare but significant complication of CS, occurring in approximately 10% of patients. Wheezing is more likely to occur in patients with metastatic carcinoid tumors. Wheezing is thought to be caused by a combination of factors, including bronchoconstriction, airway edema, and mucus hypersecretion. Carcinoid tumors can also cause other respiratory problems, such as asthma and chronic obstructive pulmonary disease (COPD) [2]. This is likely because carcinoid tumors can release a variety of substances that can irritate the airways and cause inflammation.

This case report highlights the importance of being aware of the association between carcinoid tumors and wheezing. A timely diagnosis and initiation of appropriate treatment is essential for patients with CS, as this can improve their quality of life and prolong their survival [3]. 

## Case presentation

A 61-year-old man with a past medical history of COPD and hypertension presented to the ER with new-onset angioedema, loss of consciousness, and a fall. He had a history of ER visits for wheezing and shortness of breath and was treated for a COPD exacerbation and discharged on steroids. The patient was unaware of a previous diagnosis of a mesenteric carcinoid tumor, which had been identified in 2012. After the diagnosis, he did not maintain follow-up care for this condition. Notably, the patient denied experiencing any symptoms typically associated with CS, such as skin changes or episodes of cutaneous flushing. He also confirmed that he was not taking any angiotensin-converting enzyme (ACE) inhibitors, which can be a common cause of angioedema.

**Figure 1:**
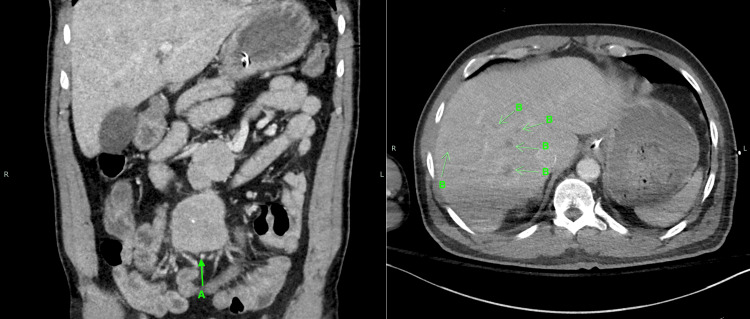
CT scan of the abdomen and pelvis showing (A) a 6.0x10.3x2.7 cm central mesenteric soft tissue mass and (B) numerous hypoattenuating lesions measuring up to 2.3 cm scattered throughout the liver

**Figure 2:**
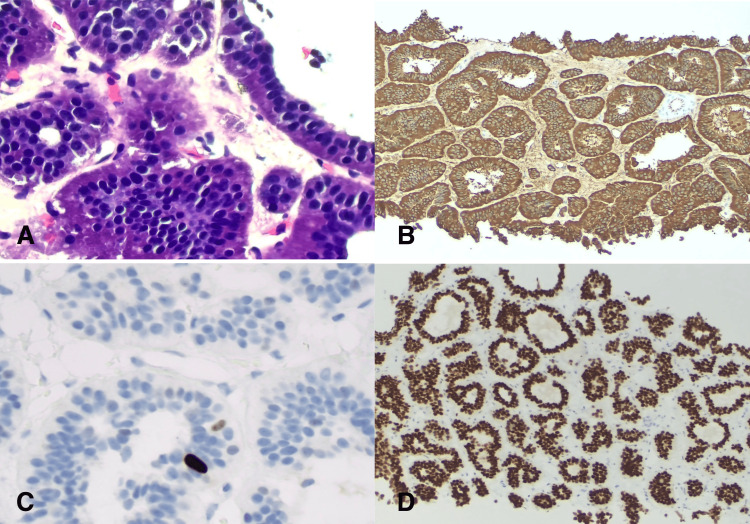
Well-differentiated neuroendocrine tumor, grade 1 of the liver: (A) The tumor cells have abundant eosinophilic and granular cytoplasm, and “salt-and-pepper“ chromatin (H&E, × 400); (B) The tumor cells are positive for chromogranin (neuroendocrine marker) (immunostain, × 40); (C) Ki-67 immunostain shows less than 1% proliferative index (immunostain, × 400); (D) The tumor cells are positive for CDX2 (immunostain, × 100)

On presentation, the patient was tachycardic with significant airway edema, lip swelling, and facial swelling. He was intubated in the field and transported to the ER. A CT scan of the abdomen and pelvis revealed a 6.0x10.3x2.7 cm central mesenteric soft tissue mass (Figure 1A) and numerous hypoattenuating lesions measuring up to 2.3 cm scattered throughout the liver (Figure 1B). A liver biopsy confirmed the diagnosis of a well-differentiated, grade 1 neuroendocrine tumor (NET) (Figure 2). Laboratory evaluation revealed elevated serum serotonin, chromogranin A, and 24-hour urine 5-hydroxyindoleacetic acid. C4 complement was normal (Table 1).

**Table 1:** Laboratory evaluation

Test	Result	Reference Range	Interpretation
Serum Serotonin	949 ng/mL	23-230 ng/mL	Elevated
Chromogranin A	590.8 ng/mL	0-101.8 ng/mL	Elevated
24-hour Urine 5-Hydroxyindoleacetic Acid (5HIAA)	152.4 mg/day	0-14.9 mg/day	Elevated
C4 Complement	29 mg/dL	12-38 mg/dL	Normal

**Figure 3:**
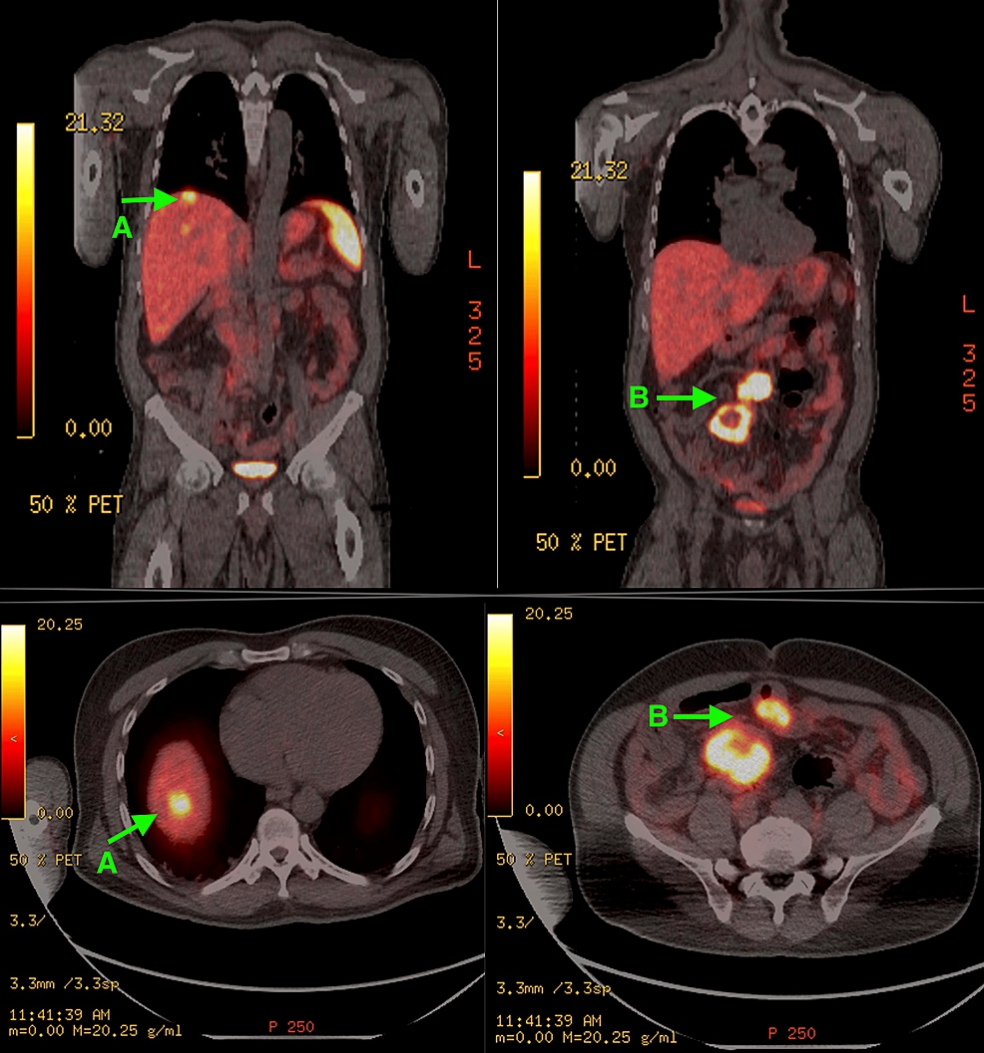
PET scan of the skull base to mid-thigh with GA-68 showing (A) Few foci of localization to the liver, compatible with metastasis; (B) Multiple foci of intense localization in several loops of small bowel in the mid-abdomen and right lower quadrant, as well as an intensively avid bilobed mass in the mesentery measuring 11 cm craniocaudal dimension

The patient was extubated the next day and discharged home on steroids with outpatient follow-up with hematology and oncology. A PET scan of the skull base to mid-thigh with GA-68 showed few foci of localization to the liver, compatible with metastasis. The majority of the liver lesions seen on the latest CT were not avid on GA68 Dotatate PET/CT. Multiple foci of intense localization were seen in several loops of small bowel in the mid-abdomen and right lower quadrant, as well as an intensively avid bilobed mass in the mesentery measuring 11 cm craniocaudal dimension (Figure 3).

The patient underwent surgery but the mass was unresectable. He was started on long-acting somatostatin analog lantreotide which inhibits the release of most of the gastrointestinal and endocrine hormones. He is currently symptom-free.

## Discussion

CS is a rare paraneoplastic syndrome characterized by the excessive secretion of serotonin and other bioactive substances from NETs. The greatest incidence of carcinoids is noted in the GI tract (67.5%), followed by the bronchopulmonary system (25.3%), and the rest are found in the thymus, liver, pancreas, ovaries, prostate, and kidneys. Within the GI tract, most carcinoid tumors occur in the small intestine (41.8%), rectum (27.4%), appendix (24.1%), and stomach (8.7%) [4]. CS is most commonly linked to midgut NETs with extensive liver metastases. However, it can also occur in patients with bronchial carcinoids and, less frequently, in those with pancreatic NETs. In cases of extensive liver metastases, a significant amount of tumor-secreted substances remain unmetabolized by liver or lung cells, entering the bloodstream and causing CS symptoms [5].

Carcinoid tumors can have the ability to secrete vasoactive peptides. Serotonin (5-hydroxytryptamine (5-HT)) production is the most prominent, especially in midgut tumors. However, 5-hydroxytryptophan (5-HTP), bradykinins, tachykinins, histamine, substance P, adrenocorticotropic hormone, and several other peptides are also reported to be produced by carcinoids. Under normal conditions, the oxidative pathway metabolizes about 99% of dietary tryptophan into nicotinic acid, and <1% is converted into 5-HTP. In carcinoid tumors, a disequilibrium of tryptophan metabolism results in the 5-hydroxylation of most of the tryptophan, with the production of large quantities of 5-HTP, 5-HT, and 5-hydroxyindolacetic acid (5-HIAA) [6]. 

While the classic presentation of CS includes flushing, diarrhea, and abdominal pain, wheezing is a relatively uncommon manifestation, occurring in approximately 10% of patients. The exact mechanism underlying wheezing in CS remains unclear. However, several possible explanations have been proposed. Serotonin, a major secretory product of NETs, can exert direct contractile effects on airway smooth muscle. Additionally, serotonin may induce the release of histamine and prostaglandins, further contributing to airway inflammation and hyperreactivity [7]. It is important to note that already-known COPD can mask CS as a COPD exacerbation. This is because both conditions can cause similar symptoms, such as wheezing, shortness of breath, and chest tightness. Additionally, patients with CS may be reluctant to report new symptoms, fearing that they will be misattributed to their COPD [8]. 

## Conclusions

This case report highlights the importance of considering CS in patients with COPD who have unexplained respiratory symptoms or whose symptoms do not respond to standard treatment. A timely diagnosis and initiation of appropriate treatment is essential for patients with CS, as this can improve their quality of life and prolong their survival.
